# Polydopamine coated manganese oxide nanoparticles with ultrahigh relaxivity as nanotheranostic agents for magnetic resonance imaging guided synergetic chemo-/photothermal therapy†
†Electronic supplementary information (ESI) available: Experimental procedures, supplementary figures and table of relaxivity of the present work and reported Mn-based nanoparticles. See DOI: 10.1039/c6sc01320a



**DOI:** 10.1039/c6sc01320a

**Published:** 2016-07-06

**Authors:** Xing Ding, Jianhua Liu, Junqi Li, Fan Wang, Yinghui Wang, Shuyan Song, Hongjie Zhang

**Affiliations:** a State Key Laboratory of Rare Earth Resource Utilization , Changchun Institute of Applied Chemistry , Chinese Academy of Sciences , Changchun 130022 , P. R. China . Email: yhwang@ciac.ac.cn ; Email: songsy@ciac.ac.cn ; Email: hongjie@ciac.ac.cn; b State Key Laboratory of Inorganic Synthesis and Preparative Chemistry , College of Chemistry , Jilin University , Changchun 130012 , P. R. China; c Department of Radiology , The Second Hospital of Jilin University , Changchun , 130022 , P. R. China

## Abstract

Mn-based nanoparticles have been regarded as a new class of probes for magnetic resonance imaging (MRI), but their low relaxivity is the major obstacle for applications *in vivo*. Herein, we designed and constructed a multifunctional nanotheranostic (FA-Mn_3_O_4_@PDA@PEG) for MRI guided combinatorial chemo-/photothermal therapy (PTT) for cancer. The ultrahigh relaxivity of 14.47 mM^–1^ s^–1^ makes the nanotheranostic an excellent contrast agent for MRI *in vitro* and *in vivo*, and provides comprehensive information for tumor diagnosis. When irradiated with an 808 nm NIR laser, FA-Mn_3_O_4_@PDA@PEG exhibits a remarkably improved and synergistic therapeutic effect compared to PTT or chemotherapy alone, providing high therapeutic efficiency and low side effects of drugs. These findings are of great interest and will inspire us to develop highly effective MRI guided synergetic chemo-/photothermal therapy for cancer treatment.

## Abstract 2

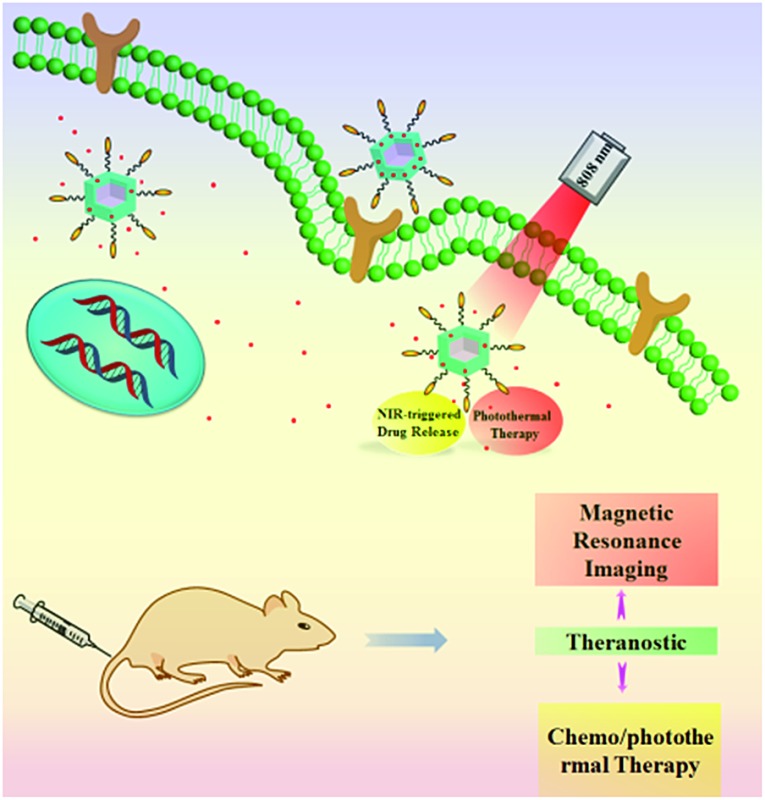
A multifunctional core/shell nanotheranostic platform was constructed which could offer MRI guided combinational chemotherapy and photothermal therapy for cancer.

## Introduction

Recent developments in the field of biomedicine have triggered significant research efforts to exploit multifunctional nanocomposites (so-called “theranostics”) that integrate imaging and therapy into a single system for imaging-guided cancer therapy since they play an important role in overcoming the limitations of traditional cancer therapy.^1^ Chemotherapy, as a general therapeutic approach for cancer, suffers greatly from limited therapeutic efficacy and severe side effects in patients. Designing multifunctional nanocomposites, which are responsive to external stimuli for on-demand chemotherapy, and are able to perform other therapeutic approaches for combined cancer therapy, is a promising strategy for addressing these problems.^2^ Lately, photothermal therapy (PTT) has received significant interest owing to its high efficiency and selectivity, minimal invasiveness, and favorable biosafety in normal tissues.^3^ Importantly, near-infrared (NIR) light could trigger the release of drugs loaded on nanocomposites, and improve the cellular uptake of the drugs. However, commonly used photothermal agents are currently inorganic nanomaterials, such as various gold nanostructures,^4^ copper sulfide nanoparticles,^5^ and carbon nanomaterials,^6^ which may cause long-term toxicity concerns in their further clinical implementation. Polydopamine (PDA), a type of melanin, has been proposed as a new generation of PTT agents with great potential for *in vivo* applications owing to its advantages of good biodegradability, no long-term toxicity, and high photothermal conversion efficiency (∼40%).^7^ Along this line, constructing theranostics based on PDA, which not only exhibits a strong photothermal effect, but also provides an active surface for loading aromatic chemotherapy drugs, could effectively improve therapeutic effectiveness and alleviate side effects.

For effective guidance for cancer therapy, an appropriate medical imaging modality is significant for identifying the tumor location and size, monitoring the biodistribution of nanocomposites and assessing the therapeutic efficacy. Magnetic resonance imaging (MRI), as a routine diagnostic tool in clinical medicine, is particularly attractive owing to its advantages of noninvasiveness, high spatial resolution and soft tissue contrast, and three-dimensional imaging.^8^ To compensate for the innate low sensitivity, a variety of contrast agents, especially positive or *T*_1_ contrast agents, are explored to increase contrast for obtaining an accurate diagnosis.^9^ Most *T*_1_ MRI contrast agents adopted currently are based on gadolinium (Gd^3+^) in the form of paramagnetic chelates or colloidal nanoparticles.^10^ Unfortunately, their potential renal toxicity, which is occasionally associated with nephrogenic system fibrosis, has aroused widespread concerns and promoted the search to find alternatives. Manganese (Mn)-based nanoparticles, especially Mn_3_O_4_ nanoparticles, have been regarded as promising alternatives owing to their lower intrinsic toxicity than Gd^3+^ ions, relatively high electronic spin, and fast water exchange rates. Nevertheless, although different size and shape Mn_3_O_4_ nanoparticles have been reported by several groups as potential *T*_1_ contrast agents,^11^ their relaxivities are usually lower than those of commercial Gd-based agents. Therefore, it is highly desirable to design and construct a theranostics nanoplatform based on Mn_3_O_4_ nanoparticles with high relaxivity for MRI guided synergetic chemo-/photothermal therapy, but it remains a great challenge.^12^

**Fig. 1:**
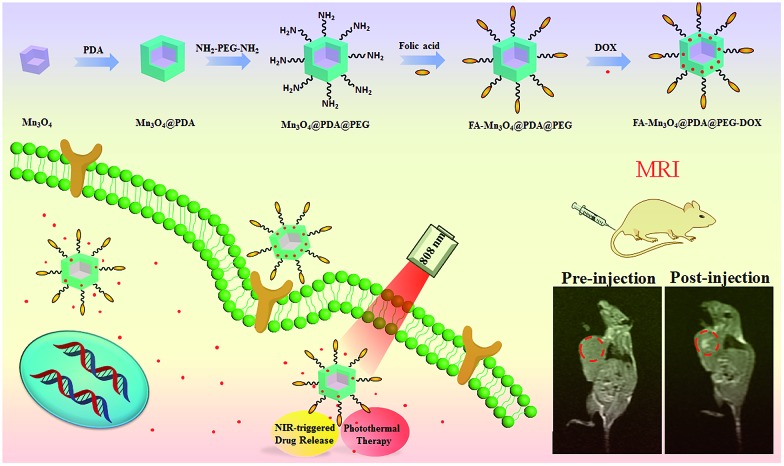
Schematic illustration of the design and synthesis of core/shell nanotheranostic Mn_3_O_4_@PDA for MRI guided synergetic chemo-/photothermal therapy.

Herein, we designed and synthesized novel multifunctional Mn_3_O_4_@PDA core/shell nanocomposites using a water-in-oil microemulsion method, then applied them as MR imaging contrast agents, photothermal agents and drug carriers for combined anticancer therapy (PTT and chemotherapy), which has not been reported until now to our best knowledge (Fig. 1). Biamino polyethylene glycol (NH_2_–PEG–NH_2_) was modified on the surface of Mn_3_O_4_@PDA for further conjugation with folic acid (FA), improving the ability to target tumors and the stability in physiological conditions. The obtained FA-Mn_3_O_4_@PDA@PEG exhibited ultrahigh longitudinal relaxivity (14.47 mM^–1^ s^–1^), which is nearly three times higher than that of the commercial Magnevist (4.96 mM^–1^ s^–1^) and the highest value reported to date over all Mn-based nanoparticles. MRI experiments were employed and the results demonstrated that FA-Mn_3_O_4_@PDA@PEG can serve well as a contrast agent *in vitro* and *in vivo*. Doxorubicin (DOX) as a model anticancer drug was loaded on the surface of PDA *via* π–π stacking and hydrogen bonding interactions, and the release could be triggered by NIR light, reducing side effects owing to the on-demand drug release. Moreover, a synergistic effect of combined photothermal therapy and chemotherapy is expected to improve the therapeutic efficiency in both *in vitro* and *in vivo* experiments.

## Results and discussion

**Fig. 2:**
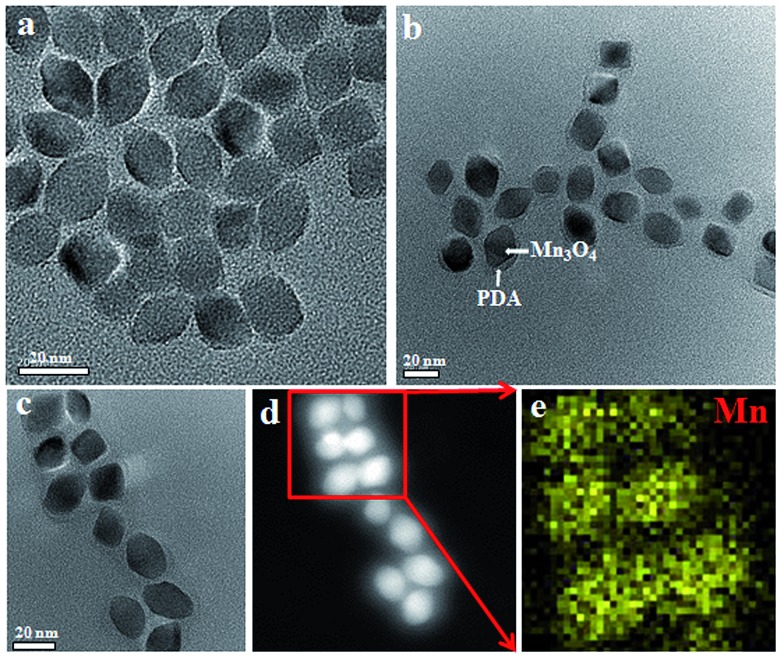
(a–c) TEM images of Mn_3_O_4_, Mn_3_O_4_@PDA, and FA-Mn_3_O_4_@PDA@PEG, (d) HAADF-STEM image of FA-Mn_3_O_4_@PDA@PEG, and (e) EDS mapping of Mn according to the image in (d).

The overall synthetic procedure for FA-Mn_3_O_4_@PDA@PEG is illustrated in Fig. 1. Briefly, Mn_3_O_4_ nanocrystals were firstly synthesized using a low-temperature method, and the average diameter was determined to be 16.8 nm using transmission electron microscopy (TEM), as presented in Fig. 2a. The crystalline nature of the as-prepared nanocrystals is further confirmed by the corresponding X-ray diffraction (XRD) pattern (Fig. S1, see ESI†), which can be indexed well to the hausmannite Mn_3_O_4_ (JCPDS 24-0734) phase.^13^ X-ray photoelectron spectroscopy (XPS) was further employed to analyze the Mn oxidation states. As shown in Fig. S2a,† in the survey spectrum, the detected peaks of Mn2p and O1s confirm the presence of Mn and O elements in the sample. The high-resolution Mn2p3/2 spectrum (Fig. S2b, see ESI†) can be resolved into two peaks at 641.2 and 642.9 eV, corresponding to the data reported for Mn^2+^ and Mn^4+^, respectively.^14^ Afterwards, Mn_3_O_4_ nanocrystals were coated with uniform PDA by a water-in-oil microemulsion method. The TEM image in Fig. 2b reveals that the PDA wrapping process on Mn_3_O_4_ was successful and the thickness of the PDA shell is approximately 4 nm. The XPS spectrum clearly shows the peaks of N1s, confirming the presence of PDA on the Mn_3_O_4_ (Fig. S3, see ESI†). With the aim of improving the accumulation of nanoparticles in tumors, NH_2_–PEG–NH_2_ was modified on the surface of the Mn_3_O_4_@PDA through a reaction between the amine group of PEG and PDA, and then the retained amine groups were available for reacting with FA *via* a carbodiimide conjugation reaction and the ether bond of the PEG chains. The amount of PEG conjugated on the surface of the Mn_3_O_4_@PDA was approximately 4%, which was assessed using thermogravimetric analysis (Fig. S4, see ESI†). The successful PEG and FA targeted functionalization is evidenced by Fourier-transform infrared spectroscopy (FT-IR) and zeta potential (Fig. S5 and S6, see ESI†). The as-obtained FA-Mn_3_O_4_@PDA@PEG is still uniform in size, and HAADF-STEM and elemental mapping analysis further confirm its core–shell structure (Fig. 2c–e).

**Fig. 3:**
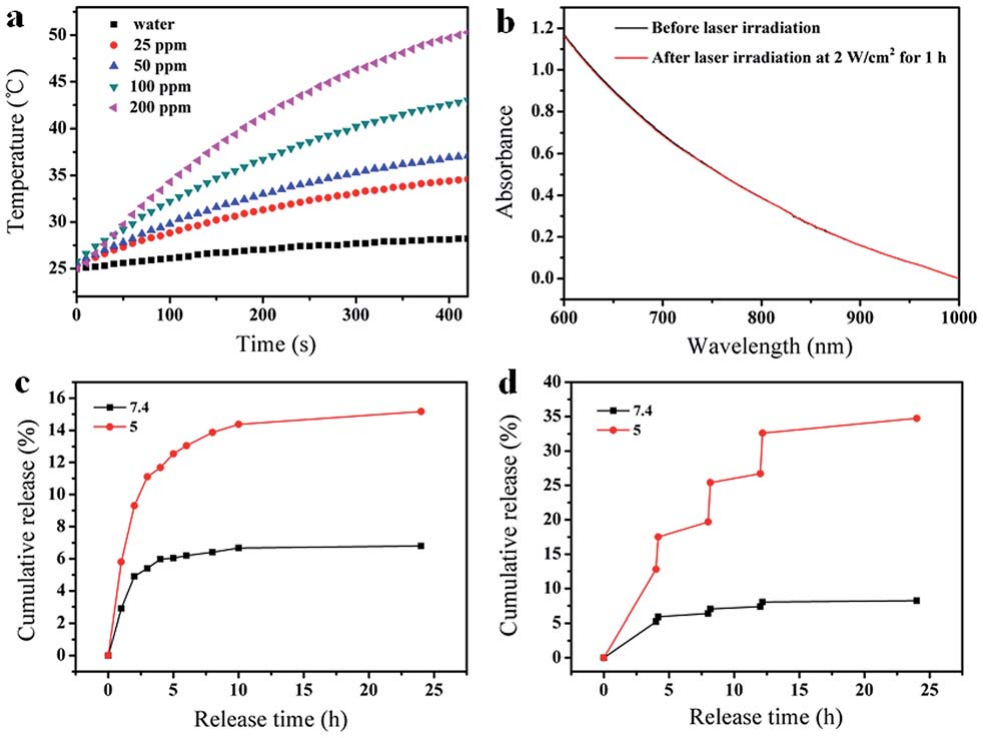
(a) Temperature elevation of water and FA-Mn_3_O_4_@PDA@PEG aqueous solutions with different Mn^2+^ concentrations as a function of time under irradiation (808 nm, 2 W cm^–2^), (b) UV-vis absorption spectra of an FA-Mn_3_O_4_@PDA@PEG dispersion in water before and after laser irradiation for 1 h (808 nm, 2 W cm^–2^), (c) DOX release from FA-Mn_3_O_4_@PDA@PEG-DOX at pH 7.4 and 5.0 at 37 °C, and (d) NIR-triggered release of DOX from FA-Mn_3_O_4_@PDA@PEG-DOX at pH 7.4 and 5.0, which were irradiated with an 808 nm NIR laser (2 W cm^–2^) for 10 min at different time points.

The FA-Mn_3_O_4_@PDA@PEG exhibits excellent colloidal stability in different dispersants including H_2_O, PBS, and 0.9% NaCl solution (Fig. S7, see ESI†). Importantly, such FA-Mn_3_O_4_@PDA@PEG shows strong NIR absorption owing to the oxidation of dopamine and the following self-polymerization process (Fig. S8, see ESI†).^7*a*^ Then, to investigate the photothermal performance, FA-Mn_3_O_4_@PDA@PEG aqueous solutions with different concentrations were exposed to an 808 nm NIR laser at 2 W cm^–2^ for 420 s. In marked contrast to pure water, FA-Mn_3_O_4_@PDA@PEG displays effective photothermal heating of the solutions (Fig. 3a). The temperatures of the solutions increase with increasing FA-Mn_3_O_4_@PDA@PEG concentration or irradiation time. After irradiation for 420 s, a rapid temperature increase of the FA-Mn_3_O_4_@PDA@PEG solution (Mn content: 200 ppm) is noted from 25 °C to 50 °C (the temperature of efficient killing of cancerous cells). Furthermore, there is no colour change or absorption decrease of the FA-Mn_3_O_4_@PDA@PEG after irradiation by an 808 nm NIR laser for 1 h (Fig. 3b and S9, see ESI†), indicating that FA-Mn_3_O_4_@PDA@PEG has high photostability. Such high photothermal efficiency and photostability makes FA-Mn_3_O_4_@PDA@PEG superior as a promising PTT agent.

Along with the capability of acting as a photothermal agent for PTT of cancer, FA-Mn_3_O_4_@PDA@PEG can load therapeutic molecules for chemotherapy. We chose an extensively used clinical chemotherapy drug DOX as a model to evaluate the carrying and releasing properties of FA-Mn_3_O_4_@PDA@PEG. After incubating with FA-Mn_3_O_4_@PDA@PEG, DOX was loaded on the surface of FA-Mn_3_O_4_@PDA@PEG at a content of 0.8014 mg mg^–1^. Such high loading capacity could be attributed to the strong π–π stacking and hydrogen bond interactions of the PDA shell with DOX since PDA has abundant phenyl, amino, and hydroxyl groups on its surface.^15^ The subsequent test of sustained release of the drug under different pH values in Fig. 3c shows that a slow drug release from the obtained FA-Mn_3_O_4_@PDA@PEG-DOX is detected at pH 7.4. By comparison, the carrier shows a relatively faster release process at pH 5, which contributes to the protonation of the amino group in the DOX molecule that offers DOX a positive charge, thus facilitating drug release under acidic conditions. Importantly, after irradiation under an 808 nm NIR laser (2 W cm^–2^, 10 min for each pulse), the release of DOX sharply increases from the FA-Mn_3_O_4_@PDA@PEG-DOX at pH 5.0 (as shown in Fig. 3d). However, there is only limited DOX release from FA-Mn_3_O_4_@PDA@PEG-DOX at pH 7.4 under the same conditions. Such pH-dependent NIR-responsive release properties show that FA-Mn_3_O_4_@PDA@PEG-DOX could be applied as a better on-demand drug delivery system which can enhance antitumor efficacy and minimize side effects of the drug.

Prior to the theranostic application of FA-Mn_3_O_4_@PDA@PEG, investigation of the cytotoxicity is an important prerequisite, so the cytotoxicity of FA-Mn_3_O_4_@PDA@PEG was assessed using a traditional MTT assay. Fig. S10† shows the *in vitro* cell viability of human breast cancer cells (MCF-7) incubated with FA-Mn_3_O_4_@PDA@PEG with different concentrations ranging from 25 to 800 μg mL^–1^ for 24 h. It can be seen that the cell viabilities are still higher than 88%, even at the highest concentration of 800 μg mL^–1^. Such findings demonstrate the near non-cytotoxicity *in vitro* for all dosages of FA-Mn_3_O_4_@PDA@PEG, which implies that FA-Mn_3_O_4_@PDA@PEG can potentially serve as a theranostic probe for simultaneous MRI and combinatorial PTT and chemotherapy of cancer.

**Fig. 4:**
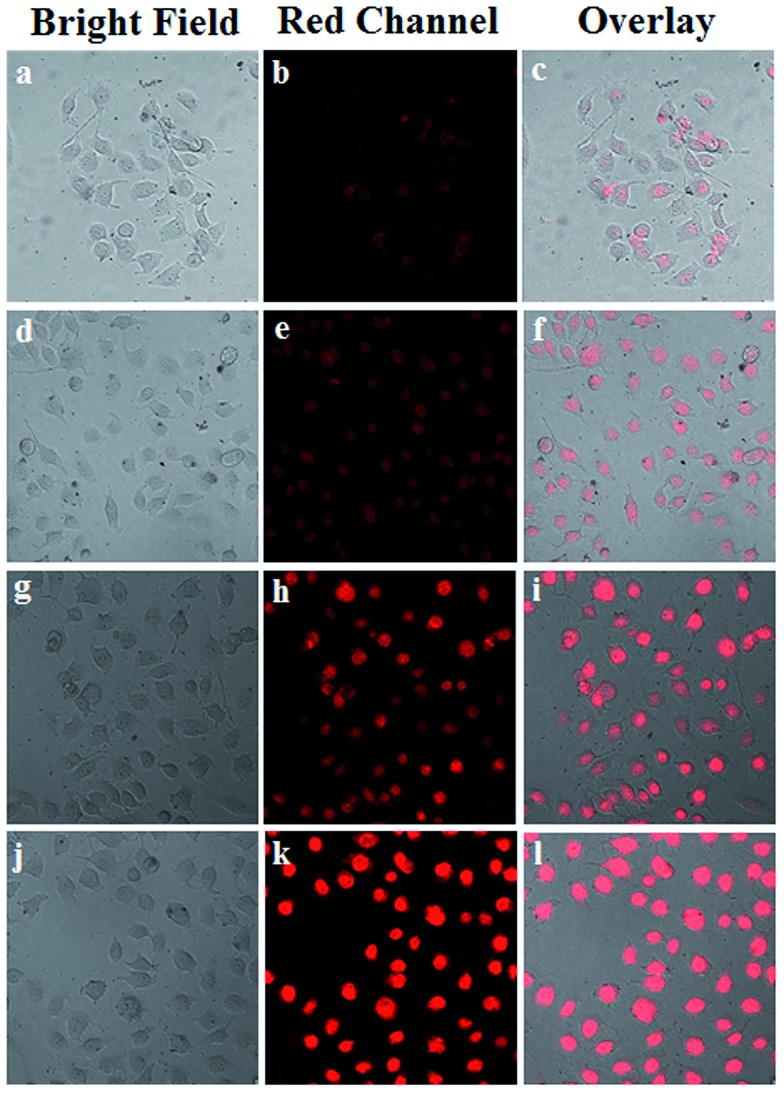
(a) CLSM images of MCF-7 cells incubated with Mn_3_O_4_@PDA@PEG-DOX for 1 h (a–c), and 3 h (d–f), and FA-Mn_3_O_4_@PDA@PEG-DOX for 1 h (g–i), and 3 h (j–l) at 37 °C.

The cellular uptake process and targeting recognition capability of FA-Mn_3_O_4_@PDA@PEG-DOX were investigated using a confocal laser scanning microscope (CLSM). Fig. 4 shows CLSM images of MCF-7 cells incubated with Mn_3_O_4_@PDA@PEG-DOX and FA-Mn_3_O_4_@PDA@PEG-DOX for 1 h and 3 h at 37 °C. Obviously, the stronger red fluorescence from the cells incubated with FA-Mn_3_O_4_@PDA@PEG-DOX than that from Mn_3_O_4_@PDA@PEG-DOX can be seen, which indicates that more folate-modified nanocomposites were uptaken specifically by MCF-7 cells. These results demonstrate that the as-prepared FA-Mn_3_O_4_@PDA@PEG-DOX is a promising candidate for targeted imaging and combinatorial PTT and chemotherapy of cancer.

**Fig. 5:**
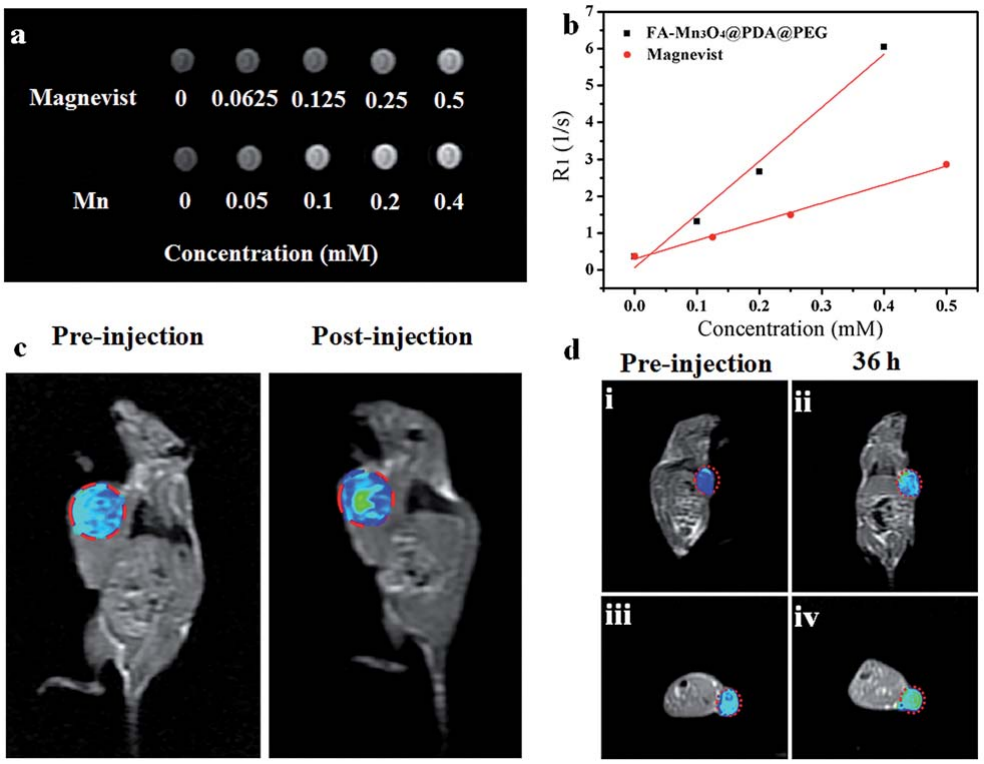
(a) *In vitro T*_1_-weighted MRI maps of FA-Mn_3_O_4_@PDA@PEG and commercial Magnevist with varied concentrations of Mn^2+^/Gd^3+^, (b) the relaxivity (*r*_1_) of FA-Mn_3_O_4_@PDA@PEG and commercial Magnevist, (c) *T*_1_-weighted MR images of a tumor-bearing Balb/c mouse: preinjection and after injection *in situ*, and (d) *in vivo T*_1_-weighted MR images of a tumor-bearing Balb/c mouse before and after intravenous injection of FA-Mn_3_O_4_@PDA@PEG. The red circles indicate the tumor sites.

Owing to their intrinsic paramagnetic properties, Mn_3_O_4_ nanoparticles are potential *T*_1_-MRI contrast agents by accelerating the longitudinal relaxation of water protons. To evaluate the capacity of FA-Mn_3_O_4_@PDA@PEG as a *T*_1_-MRI contrast agent for diagnostics, we investigated the magnetic resonance signals of FA-Mn_3_O_4_@PDA@PEG and Magnevist (a traditional MRI contrast agent currently used in clinic) using a 1.2 T MRI system. Fig. 5a displays the *T*_1_-weighted relaxivity of FA-Mn_3_O_4_@PDA@PEG and shows that the MRI contrast is enhanced by increasing the concentration of Mn. The longitudinal relaxivity value (*r*_1_) is calculated to be 14.47 mM^–1^ s^–1^ from the slope of the concentration-dependent relaxation 1/*T*_1_ (Fig. 5b). This value is nearly three times higher than that of Magnevist (4.96 mM^–1^ s^–1^) under the same testing conditions, which may be ascribed to the greater number of free sites of FA-Mn_3_O_4_@PDA@PEG for water ligation. Most importantly, it is the highest value reported to date for Mn-based NPs (Table S1, see ESI†), which reduces the concentration required for contrast agent detection by MRI. This result further confirms that FA-Mn_3_O_4_@PDA@PEG could operate as an excellent positive MRI contrast agent.

To assess the *in vivo* MR imaging, we intratumorally injected FA-Mn_3_O_4_@PDA@PEG into a tumor-bearing mouse, and used a 3.0 T human MRI scanner. As shown in Fig. 5c, the significantly enhanced MRI signal can be clearly observed in the injection area. Then, FA-Mn_3_O_4_@PDA@PEG was intravenously administered into a tumor-bearing mouse to further evaluate its targeting MRI capacity *in vivo*. As shown in Fig. 5d, different from the homogeneous and slightly dark image before injection, the whole tumor area becomes brighter after 36 h injection. Such results demonstrate that a large amount of FA-Mn_3_O_4_@PDA@PEG accumulates in the tumor owing to the active targeting ability of the FA and the enhanced permeability and retention (EPR) effect occurring in the vessels of the cancer tissues. Importantly, the enhancement of the MRI signals in the tumor is capable of continuing for 36 hours, which provides adequate time for guiding the following treatment. The active targeting and high MRI contrast ability make FA-Mn_3_O_4_@PDA@PEG a promising candidate for accurate cancer diagnosis and locating the tumor site to guide the external NIR laser irradiation for photothermal therapy and controlling the drug release on-demand.

**Fig. 6:**
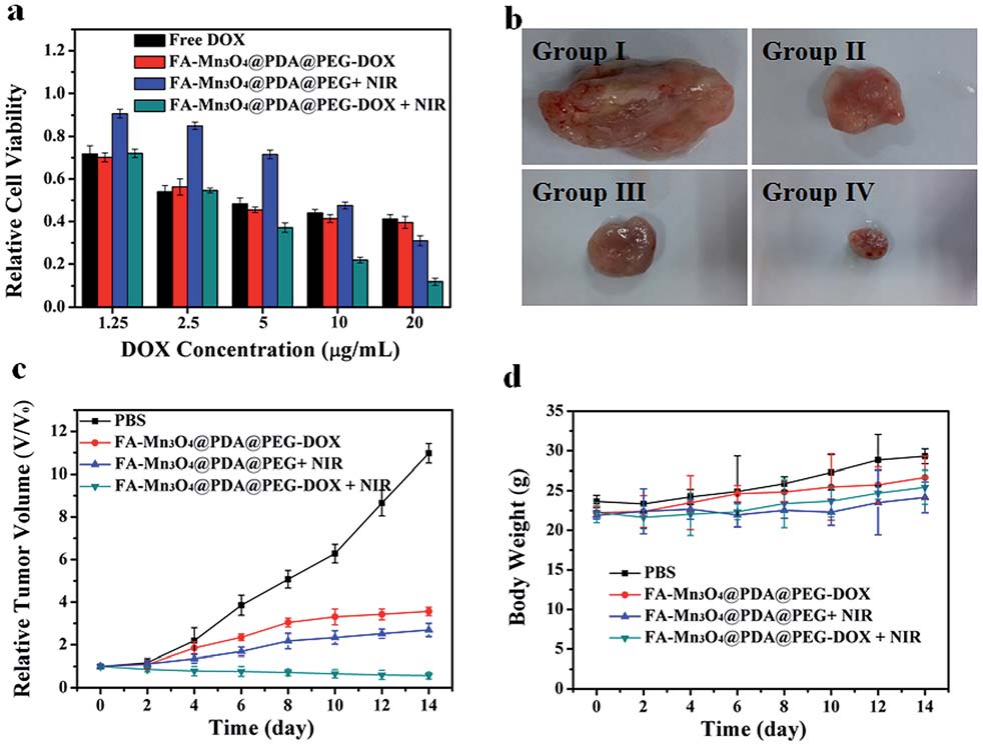
(a) Cell viability of MCF-7 cells incubated with free DOX, FA-Mn_3_O_4_@PDA@PEG-DOX, FA-Mn_3_O_4_@PDA@PEG + NIR, and FA-Mn_3_O_4_@PDA@PEG-DOX + NIR. (b) Photographs of excised tumors from representative euthanized mice. (c) Relative tumor volume and (d) body weight of mice after treatment with a PBS solution as control, FA-Mn_3_O_4_@PDA@PEG-DOX, FA-Mn_3_O_4_@PDA@PEG + NIR, and FA-Mn_3_O_4_@PDA@PEG-DOX + NIR.

Inspired by the properties of high photothermal efficiency and NIR triggered controllable drug release, we further investigated the antitumor effects of FA-Mn_3_O_4_@PDA@PEG-DOX *in vitro* and *in vivo*. MCF-7 cells were treated with free DOX, FA-Mn_3_O_4_@PDA@PEG, and FA-Mn_3_O_4_@PDA@PEG-DOX for 10 min with or without NIR laser irradiation. As shown in Fig. 6a, after exposure to the NIR laser for 10 min, FA-Mn_3_O_4_@PDA@PEG-DOX exhibits the highest statistically cytotoxic effect at all the tested concentrations compared to the chemotherapy and photothermal therapy alone. For example, when DOX concentration is 20 μg mL^–1^, the cell viability for FA-Mn_3_O_4_@PDA@PEG-DOX + NIR is remarkably reduced to 11%, which is evidently lower than that of free DOX, FA-Mn_3_O_4_@PDA@PEG-DOX alone, and FA-Mn_3_O_4_@PDA@PEG + NIR. The remarkably improved therapeutic effect may be attributed to the photothermal effect, which can not only kill the cancer cells but also effectively enhance the sensitivity of the delivery and release of DOX into cells for improved chemotherapy for cancer.

To shed more light on the combined therapeutic effects of FA-Mn_3_O_4_@PDA@PEG-DOX, we further investigated the effectiveness of tumor inhibition *in vivo* using H22 tumor-bearing mice. The H22 (murine hepatocarcinoma) xenograft model was established by injecting H22 cancer cells into the right axilla of female Balb/c mice. The mice bearing tumors were randomly divided into four groups (group I to IV) and treated with PBS (as control), FA-Mn_3_O_4_@PDA@PEG-DOX, FA-Mn_3_O_4_@PDA@PEG + NIR irradiation, and FA-Mn_3_O_4_@PDA@PEG-DOX + NIR irradiation, respectively. The tumor dimensions were tracked every 2 days with a caliper for 14 days. After treatment, the tumors were isolated from the different groups of mice and weighed. As shown in Fig. 6b and c, the tumor growth of group II and group III was only slightly delayed compared to group I, indicating that either chemotherapy or photothermal therapy by itself exhibited only partial inhibitory effects. In contrast, when the mice were injected with FA-Mn_3_O_4_@PDA@PEG-DOX and then exposed to 808 nm NIR laser triggered photothermal therapy and drug release, the tumor growth was remarkably inhibited with tumor growth inhibition (TGI) of 94.81%, demonstrating that combination chemotherapy and PTT exhibited the optimal therapeutic efficacy. Moreover, the body weight of all groups did not decrease with the time prolonged, indicating no significant acute toxicity of our theranostic agent. Such results clearly confirm that FA-Mn_3_O_4_@PDA@PEG-DOX has potential applications for *in vivo* combinatorial chemotherapy and PTT for cancer.

## Conclusions

In summary, a multifunctional core/shell FA-Mn_3_O_4_@PDA@PEG nanotheranostic has been successfully constructed for MRI guided combinatorial chemo-/photothermal therapy for cancer. The ultrahigh relaxivity of 14.47 mM^–1^ s^–1^ makes the nanotheranostic an excellent contrast agent for MRI *in vitro* and *in vivo*, and provides comprehensive information for tumor diagnosis. The PDA shell not only is employed as a high photothermal conversion agent for PTT and aromatic anticancer drug carrier, but also endows the nanotheranostic with robust biocompatibility owing to its natural characteristics. Moreover, the PTT and on-demand drug release are simultaneously triggered by 808 nm NIR laser irradiation, which effectually enhances the anticancer effect and reduces adverse side effects of drugs. Compared with chemotherapy or photothermal treatment alone, the combined treatment shows a synergistic effect, resulting in higher therapeutic efficacy for *in vitro* and *in vivo* cancer therapy. Therefore, this multifunctional nanocomposite is expected to be employed as a powerful theranostics platform for MRI guided therapeutics allied with significant therapeutic effectiveness but low side effects in future oncotherapy.

## References

[cit1] Greco F., Vicent M. (2009). Adv. Drug Delivery Rev..

[cit2] Liu J., Wang C., Wang X., Wang X., Cheng L., Li Y., Liu Z. (2015). Adv. Funct. Mater..

[cit3] Lal S., Clare S. E., Halas N. J. (2008). Acc. Chem. Res..

[cit4] Xia Y., Li W., Cobley C. M., Chen J., Xia X., Zhang Q., Yang M., Cho E. C., Brown P. K. (2011). Acc. Chem. Res..

[cit5] Tian Q., Tang M., Sun Y., Zou R., Chen Z., Zhu M., Yang S., Wang J., Hu J. (2011). Adv. Mater..

[cit6] Yang K., Zhang S., Zhang G., Sun X., Lee S.-T., Liu Z. (2010). Nano Lett..

[cit7] Liu Y., Ai K., Liu J., Deng M., He Y., Lu L. (2013). Adv. Mater..

[cit8] Feshitan J. A., Vlachos F., Sirsi S. R., Konofagou E. E., Borden M. A. (2012). Biomaterials.

[cit9] Wang S. H., Shi X., Van Antwerp M., Cao Z., Swanson S. D., Bi X., Baker J. R. (2007). Adv. Funct. Mater..

[cit10] Caravan P. (2006). Chem. Soc. Rev..

[cit11] Huang C. C., Khu N. H., Yeh C. S. (2010). Biomaterials.

[cit12] Nafiujjaman M., Nurunnabi M., Kang S., Reeck G. R., Khand H. A., Lee Y. (2015). J. Mater. Chem. B.

[cit13] Tan Y., Xu C., Chen G., Fang X., Zheng N., Xie Q. (2012). Adv. Funct. Mater..

[cit14] Di Castro V., Polzonetti G. (1989). J. Electron Spectrosc. Relat. Phenom..

[cit15] Liu F., He X., Lei Z., Liu L., Zhang J., You H., Zhang H., Wang Z. (2015). Adv. Healthcare Mater..

