# Supplementary material for: Polydopamine coated manganese oxide nanoparticles with ultrahigh relaxivity as nanotheranostic agents for magnetic resonance imaging guided synergetic chemo-/photothermal therapy
Source: Chem Sci. 2016 Jul 6;7(11):6695–700. doi: 10.1039/c6sc01320a (PMC5355856; doi:10.1039/c6sc01320a)
Supplement: Supplementary file 1 [file SC-007-C6SC01320A-s001.pdf]

Electronic supplementary information (ESI)

Polydopamine coated manganese oxide nanoparticles with ultrahigh relaxivity as nanotheranostic agents for magnetic resonance imaging guided synergetic chemo-/photothermal therapy

Xing Ding,[a,b] Jianhua Liu,[b,c] Junqi Li,[a] Fan Wang,[a] Yinghui Wang [a,] * Shuyan Song,[a,] * and Hongjie Zhang [a,] *

a. State Key Laboratory of Rare Earth Resource Utilization, Changchun Institute of Applied Chemistry, Chinese Academy of Sciences, Changchun 130022, P. R. China. E-mail: yhwang@ciac.ac.cn; songsy@ciac.ac.cn; hongjie@ciac.ac.cn

b. State Key Laboratory of Inorganic Synthesis and Preparative Chemistry, College of Chemistry, Jilin University, Changchun 130012, P. R. China

c. Department of Radiology, the Second Hospital of Jilin University, Changchun, 130022, P. R. China

1. Experimental Section

1.1 Applied Chemicals

Manganese(II) acetate and xylene were purchased from Beijing Chemical Regent Co., Ltd. Oleylamine (>90%), oleic acid (OA, >90%), dopamine hydrochloride and folic acid (FA, >97%) were purchased from Aladdin. Igepal CO-520, N-hydroxysuccinimide (NHS) and 1-ethyl-3-(3-dimethylaminopropyl) carbodiimide hydrochloride (EDC) were purchased from Sigma-Aldrich. Biamino polyethylene glycol (NH_2-PEG-NH_2) was purchased from Sunbio Inc. All above chemicals were used directly without further purification.

1.2 Synthesis of FA targeted Mn_3O_4@PDA nanocomposites.

1.2.1. Synthesis of Mn_3O_4 NPs nanoparticles

Mn_3O_4 nanoparticles were synthesized following previously reported method.[R1] In details, 15 mL of xylene was added to a 50 mL flask containing 0.17 g of manganese(II) acetate, 0.67 g of oleylamine and 0.14 g of OA. Then the solution was slowly heated to 90 °C with vigorous magnetic stirring. 1 mL of deionized water was added and the solution was further heated at 90 °C for 1.5 h. The Mn_3O_4 NPs were collected by centrifugation, washed with ethanol for several times, and dispersed in 20 mL cyclohexane.

1.2.2. Synthesis of PEG-functionalized Mn_3O_4@PDA nanocomposites

PEG-functionalized Mn_3O_4@PDA nanocomposites were synthesized following the previous report.[R2] The obtained Mn_3O_4 nanoparticles in 10 mL cyclohexane were added to a 50 mL flask containing 0.65 mL Igepal CO-520 and 5 mL cyclohexane.

After stirred for 30 min, 75 μL ammonia solution (25 %) was injected into the flask, then sonicated for 10 min at room temperature and stirred for another 30 min. Next, 20 μL dopamine hydrochloride aqueous solution (10 wt%) was injected into the solution at a rate of 5 μL min^{-1}. After stirred for another 24 h, Mn_3O_4@PDA nanocomposites were precipitated by addition of ethanol, collected by centrifugation, washed with deionized water several times. The obtained nanoparticles dispersed in 5 mL deionized water were added into 30 mL Tris buffer (pH 8.5) containing 100 mg NH_2-PEG-NH_2. After stirred for 12 h, The PEG-functionalized Mn_3O_4 nanocomposites (Mn_3O_4@PDA@PEG) were collected by centrifugation, washed with deionized water for several times, and dispersed in 10 mL deionized water.

1.2.3. Synthesis of FA-Mn_3O_4@PDA@PEG nanocomposites

FA-Mn_3O_4@PDA@PEG nanocomposites were synthesized according to the literature.[R3] In details, 10 mg FA in 10 mL of PBS (pH 7.4) was stored in refrigerator at 4 °C for 20 min. Then EDC (8 mg) and NHS (6 mg) were added into the cooled solution immediately, and the solution was kept at room temperature with vigorous stirring for 8 h. After that, the obtained Mn_3O_4@PDA-PEG NPs in 10 mL deionized water were added dropwise into the solution and stirred for another 16 h at room temperature. The FA-Mn_3O_4@PDA@PEG were collected by centrifugation.

1.3 Drug loading and releasing

DOX loading onto FA-Mn_3O_4@PDA@PEG was done by mixing DOX (3mL, 1mg/mL) with FA-Mn_3O_4@PDA@PEG in Tris buffer (pH 8.5). After being stirred at 37 ℃ for 24 h in the dark, FA-Mn_3O_4@PDA@PEG-DOX was separated by

centrifugation, gently washed with PBS for 3 times. All the washing supernatant solutions were collected for measuring the DOX loading content by UV−vis measurement. For investigating the drug release, FA-Mn_3O_4@PDA@PEG-DOX was dispersed in 5 mL of different buffer solutions (pH 7.4 or pH 5) and sealed in a dialysis bag (molecular weight cutoff = 8000). The dialysis bag was submerged in 20 mL of respective buffer solutions and stirred at 37 °C for 24 h. The released DOX in the buffer was collected at selected time intervals and analyzed by UV-vis spectroscopy. As for the NIR triggered release, the samples were irradiated with an 808 nm NIR laser (2 W cm^{-2}) for 10 min at predetermined time intervals. The released DOX was collected for before and after 808 nm NIR laser stimulation, respectively.

1.4 Cytotoxicity assay

The human breast cancer cell line MCF-7 cell, provided by the Institute of Biochemistry and Cell Biology, SIBS, CAS (China), were cultured in RPMI-1640 culture medium containing 10% FBS and 1% penicillin/streptomycin at 37 ℃ under 5% CO_2. The cells were seeded in 96-well plates (5×10^4 cells per well) for 24 h, and then incubated with various concentrations of FA-Mn_3O_4@PDA@PEG for 24 h. Then 10 μL MTT solution (5 mg/mL MTT in PBS, pH 7.4) was added to each well and the plate was incubated for an additional 4 h. After removing the medium, the wells were washed by PBS, and then the intracellular formazan crystals were extracted into 100 μL DMSO. The absorbance was recorded at 490 nm by a plate reader, and the cell viability could be calculated from the average value of six parallel wells.

1.5 Cellular uptake

Cellular uptake by MCF-7 cells was examined using confocal laser scanning microscope (CLSM). MCF-7 cells were plated on 14 mm glass coverslips under 100% humidity and allowed to adhere for 24 h. The cells were incubated with FA-Mn_3O_4@PDA@PEG-DOX and Mn_3O_4@PDA@PEG-DOX at 37 ℃ for different times, respectively. Prior to imaging, the coverslips were washed three times with PBS. Confocal imaging of cells was performed with a Olympus FV1000 laser scanning microscope.

1.6 Chemotherapy and PTT treatments in vitro and in vivo

MCF-7 cells were seeded in 96-well plates (5×10^4 cells per well) for 24 h, and then incubated with various concentrations of FA-Mn_3O_4@PDA@PEG, FA-Mn_3O_4@PDA@PEG-DOX, and free DOX. After 4 h of incubation, the cells incubated with FA-Mn_3O_4@PDA@PEG, FA-Mn_3O_4@PDA@PEG-DOX were washed twice with PBS and irradiated using 808 nm NIR laser at a power density of 2 W cm^{-2} for 10 min. Then, the cells were allowed to incubate for another 24 h. Thereafter, the standard MTT assay was carried out to determine the cell viabilities.

All animal studies were performed in accordance with institutional and national guidelines. Female Balb/c mice (18-20 g) were purchased from Center for Experimental Animals, Jilin University (Changchun, China). The tumors were established by subcutaneous injection of H22 cells (murine hepatocarcinoma cell lines) in the right axilla of each mouse. The tumors were allowed to grow for around 4 days to reach the size of around 6-10 mm. The tumor-bearing mice were randomized into

four groups (n = 5, each group) and were treated by intratumoral injection with PBS (as control), FA-Mn_3O_4@PDA@PEG + NIR irradiation, FA-Mn_3O_4@PDA@PEG-DOX and FA-Mn_3O_4@PDA@PEG-DOX + NIR irradiation, respectively. The body weights and tumor sizes were recorded every 2 days after the treatment.

1.7 In vitro and in vivo T_1 weighted MR imaging

The in vitro MR imaging experiments were performed in a 1.2 T MRI magnet (Shanghai Niumai Corporation Ration NM120-Analyst). FA-Mn_3O_4@PDA@PEG was dispersed in water at various Mn concentrations (by ICP-MS measurement). T_1 was acquired using inversion recovery sequence. T_1 measurements were performed using a nonlinear fit to changes in the mean signal intensity within each well as a function of repetition time (TR). Finally, the r_1 relaxivity values were determined through the curve fitting of $1/T_1$ relaxation time (s^{-1}) vs. the Mn concentration (mM).

In vivo MR imaging experiments were performed on a 3.0 T clinical MRI instrument (Siemens Medical System). The tumor bearing mouse was anesthetized with 10 % chloral hydrate by intraperitoneal injection. The mouse was scanned before and after intratumoral injection of FA-Mn_3O_4@PDA@PEG nanoparticles. For intravenous injection, FA-Mn_3O_4@PDA@PEG nanoparticles were injected into the mouse. MRI images were acquired at timed intervals.

Table S1. Comparison of the relaxivity of present work with reported Mn-based nanoparticles.

Materials	Core element	r_1 (mM^{-1} s^{-1})	Field (T)	Ref.
**FA-Mn_3O_4@PDA@PEG**	Mn_3O_4	14.47	1.2	This work
**HMnO@mSiO_2**	MnO	0.99	11.7	R4
**MnO@PEG-phospholipid**	MnO	0.11	11.7	R4
**MnO@mSiO_2**	MnO	0.65	11.7	R4
**MnO@dSiO_2**	MnO	0.08	11.7	R4
**Mn_3O_4 nanospheres**	Mn_3O_4	1.31	3	R5
**Mn_3O_4 nanoplates**	Mn_3O_4	2.06	3	R5
**Mn_3O_4 nanocubes**	Mn_3O_4	1.08	3	R5
**MnO nanoplates**	MnO	5.5	3	R6
**Mn_3O_4@SiO_2**	Mn_3O_4	0.47	3	R7
**WMON**	MnO	0.21	3	R8
**HMnO**	Mn_3O_4	1.42	3	R8
**Mn-NMOFs**	MnO	4	9.4	R9
**MnO**	MnO	0.12	3	R10
**Mn-MSNs**	MnO(Mn_3O_4)	2.28	3	R11
**Mn_3O_4**	Mn_3O_4	8.26	3	R12
**HAS-MNOP**	MnO	1.97	7	R13

**MnO-TETT**	MnO	4.84	7	R14
**Mn_3O_4 nanospheres**	Mn_3O_4	2.38	1.5	R15
**tetrahedral Mn_3O_4**	Mn_3O_4	1.08	1.5	R15
**Mn_3O_4@SiO_2-PEG–Apt**	Mn_3O_4	0.53	0.5	R16
**PEOMSNs**	MnO	1.17	7	R17
**MnOx-HMCNs**	MnO	10.05	3	R18
**PTNPs**	MnO	2.4	7	R19
**MONs**	MnO	2.58	7	R20
**GQD–PDA–Mn_3O_4**	Mn_3O_4	3.45	3	R21

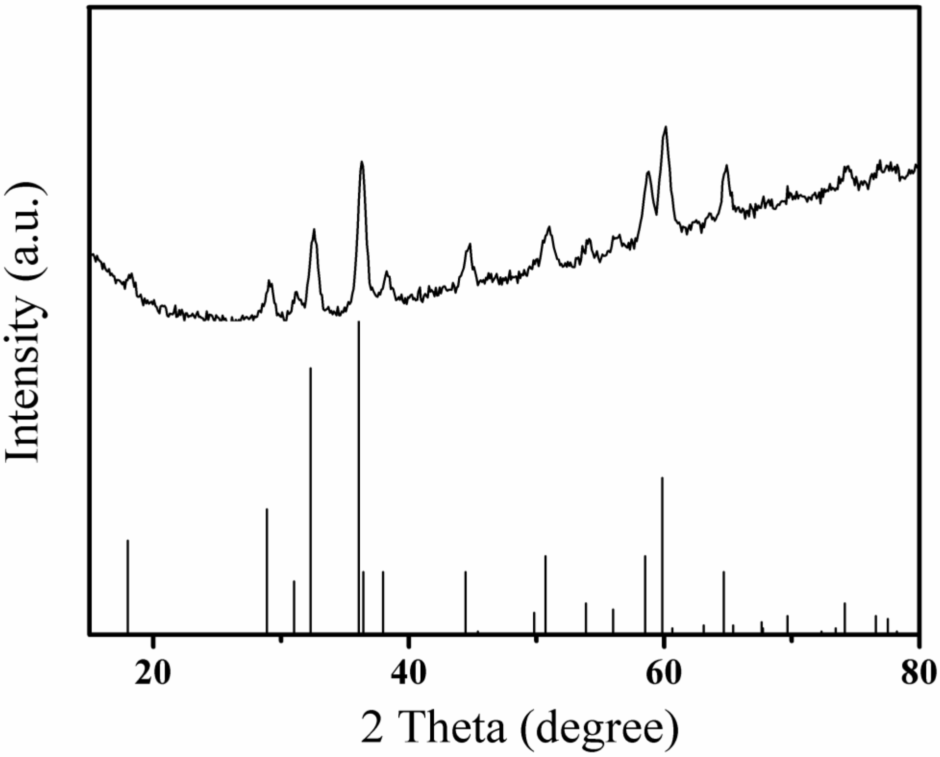

Figure S1. The X-ray diffraction pattern of Mn_3O_4, and the standard pattern of hausmannite Mn3O4 is given for reference (JCPDS card 24-0734).

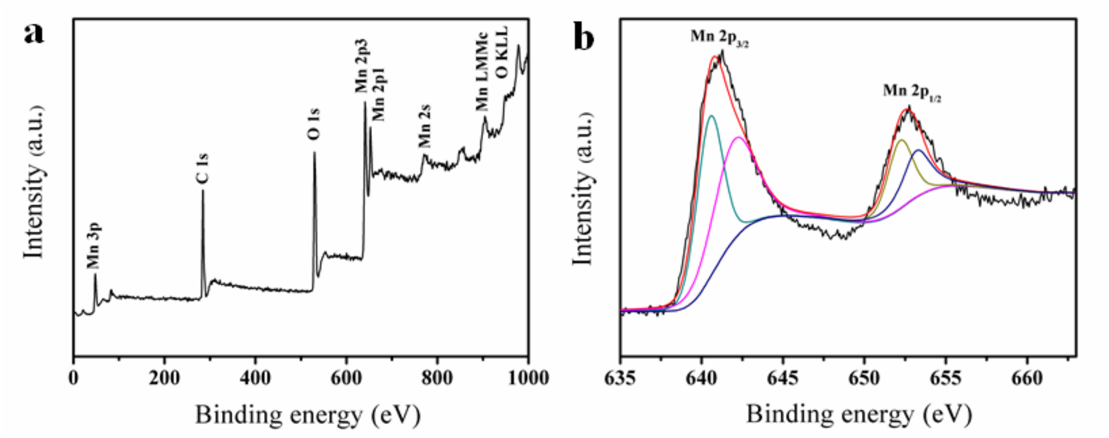

Figure S2. XPS spectra for the Mn_3O_4: (a) the survey spectrum and the high resolution spectra for (b) Mn 2p.

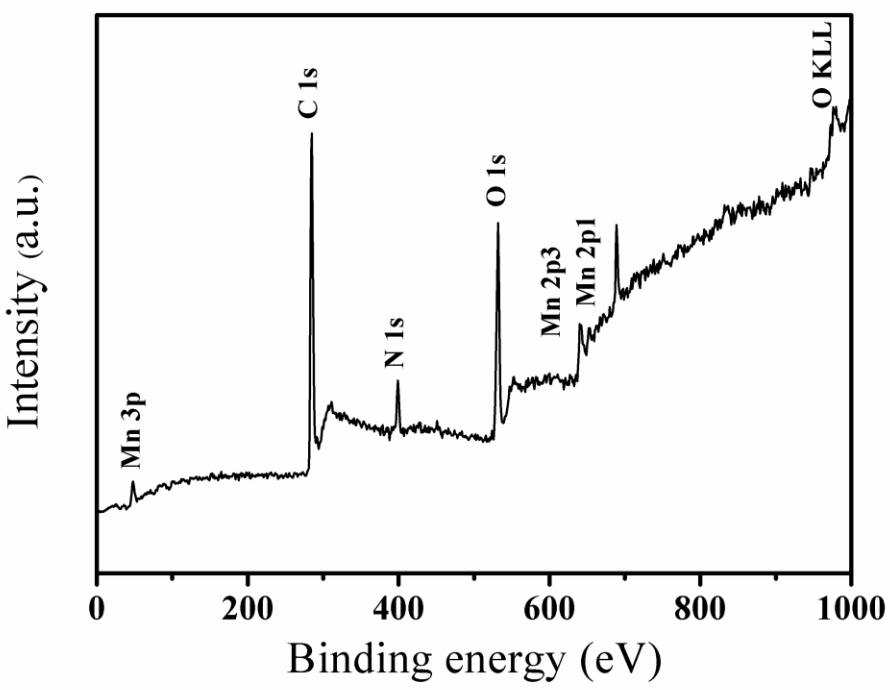

Figure S3. XPS spectra for the Mn_3O_4@PDA.

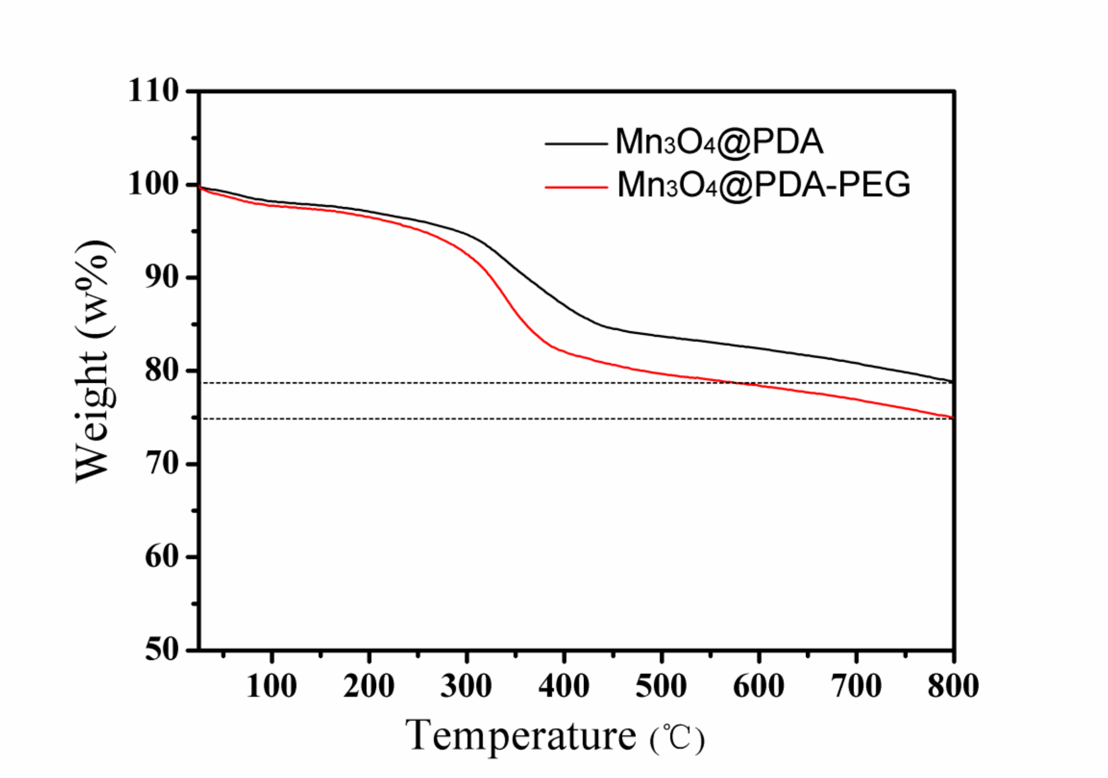

Figure S4. TGA curve of Mn_3O_4@PDA nanoparticles and Mn_3O_4@PDA-PEG nanoparticles.

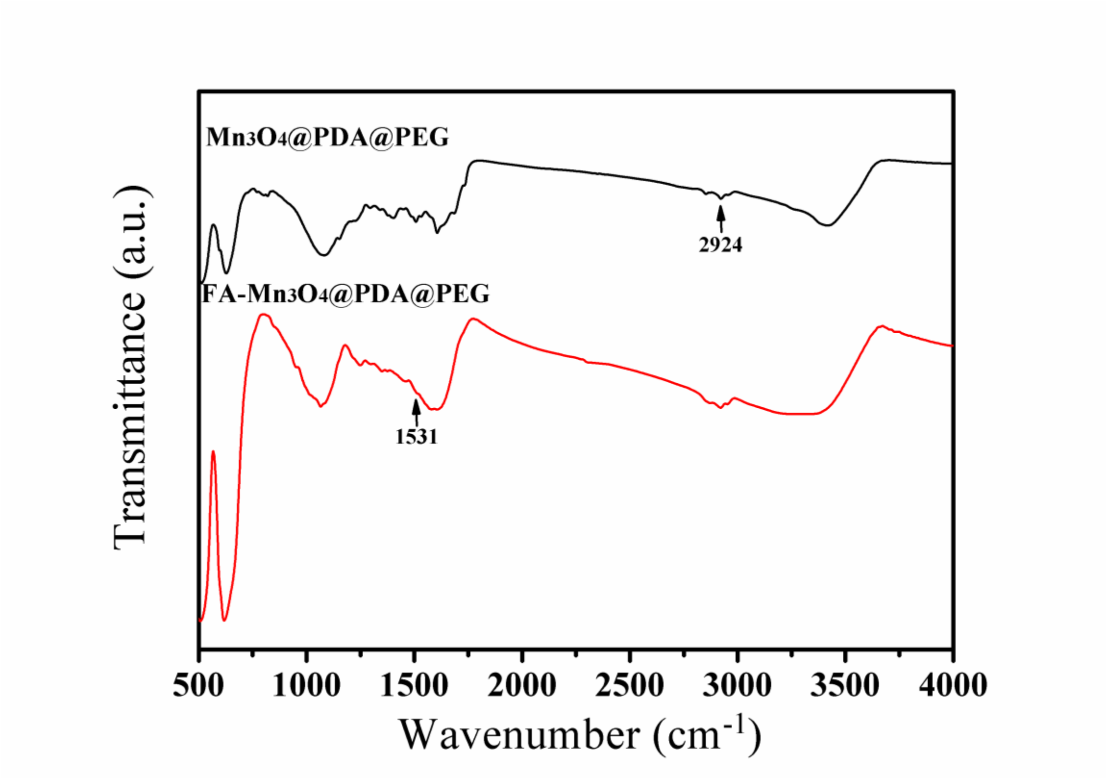

Figure S5. FT-IR spectra of Mn_3O_4@PDA@PEG and FA-Mn_3O_4@PDA@PEG, respectively.

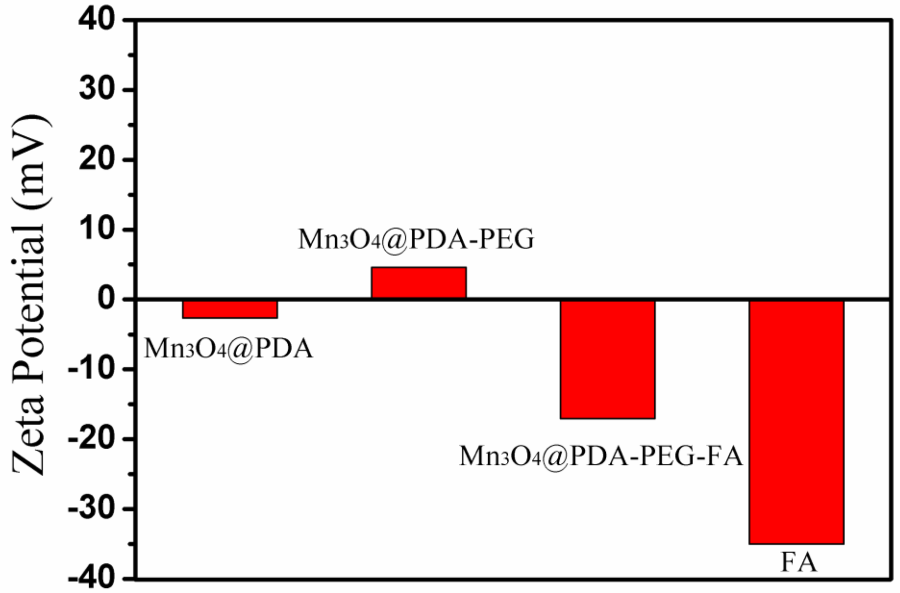

Figure S6. Zeta potential of Mn_3O_4@PDA, Mn_3O_4@PDA-PEG, Mn_3O_4@PDA-PEG-FA, and FA.

The Zeta potential of Mn_3O_4@PDA, Mn_3O_4@PDA-PEG, Mn_3O_4@PDA-PEG-FA, and FA were measured to be -2.65, 4.62, -17.03, and -35.00 mV, respectively, as shown in Figure 2. Because PDA has numerous functional groups such as the catechol groups on the surface, the Zeta potential of Mn_3O_4@PDA was negative. After being modified by NH_2-PEG-NH_2, the surface charge of Mn_3O_4@PDA-PEG became positive. After modifying the FA on the surface of Mn_3O_4@PDA-PEG, the Zeta potential changed to negative, but it was higher than that of FA. This phenomenon was on account of that the amino groups on the surface of Mn_3O_4@PDA-PEG were partly reacted with carboxyl from FA molecules.

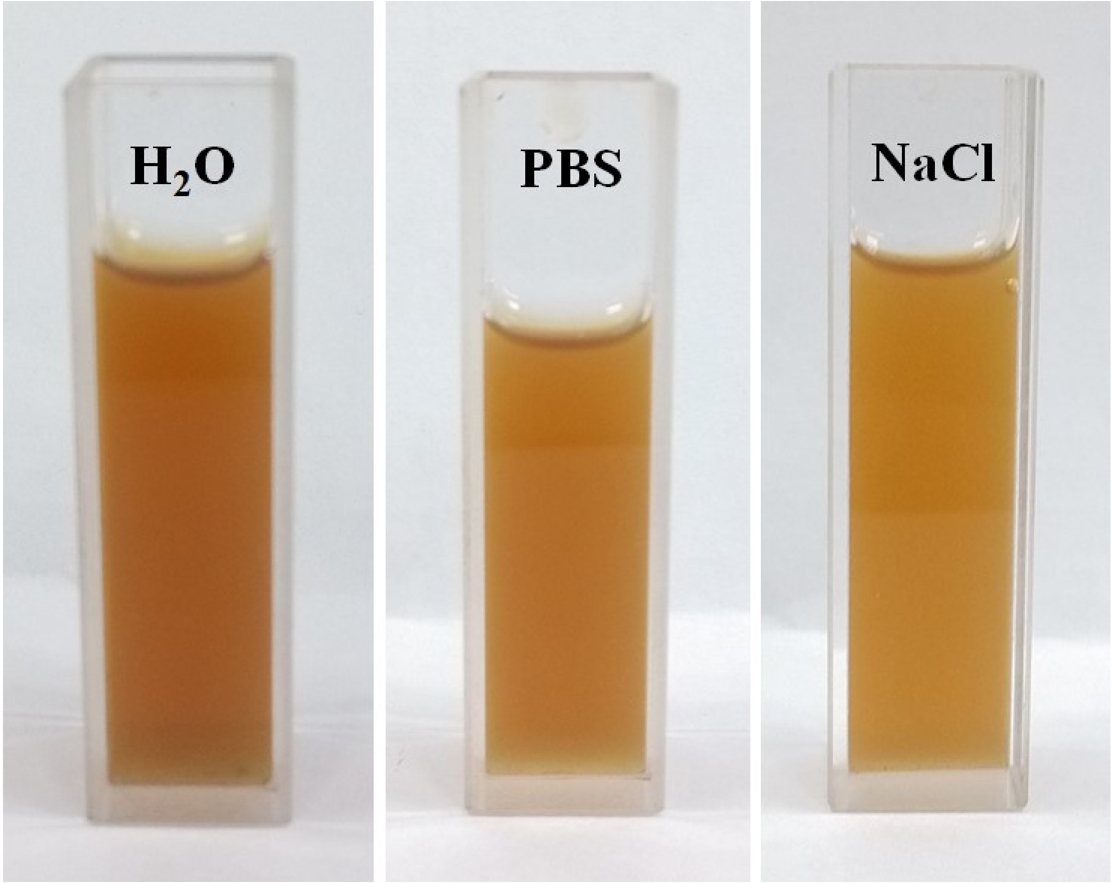

Figure S7. The photographs of FA-Mn_3O_4@PDA@PEG dispersion in water, PBS and 0.9% NaCl solution.

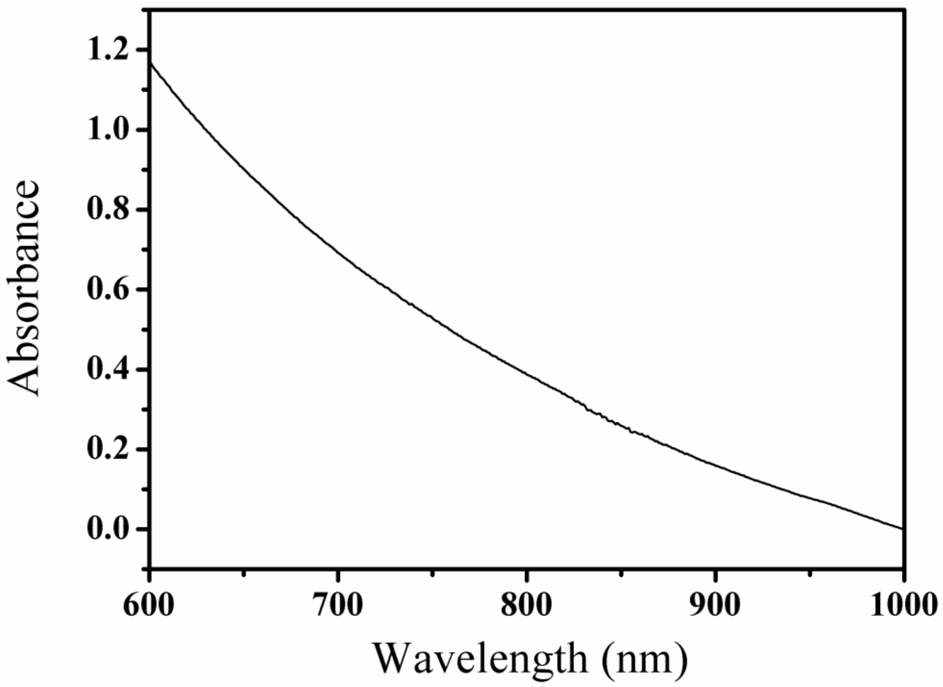

Figure S8. UV–vis absorption spectrum of FA-Mn_3O_4@PDA@PEG aqueous solution.

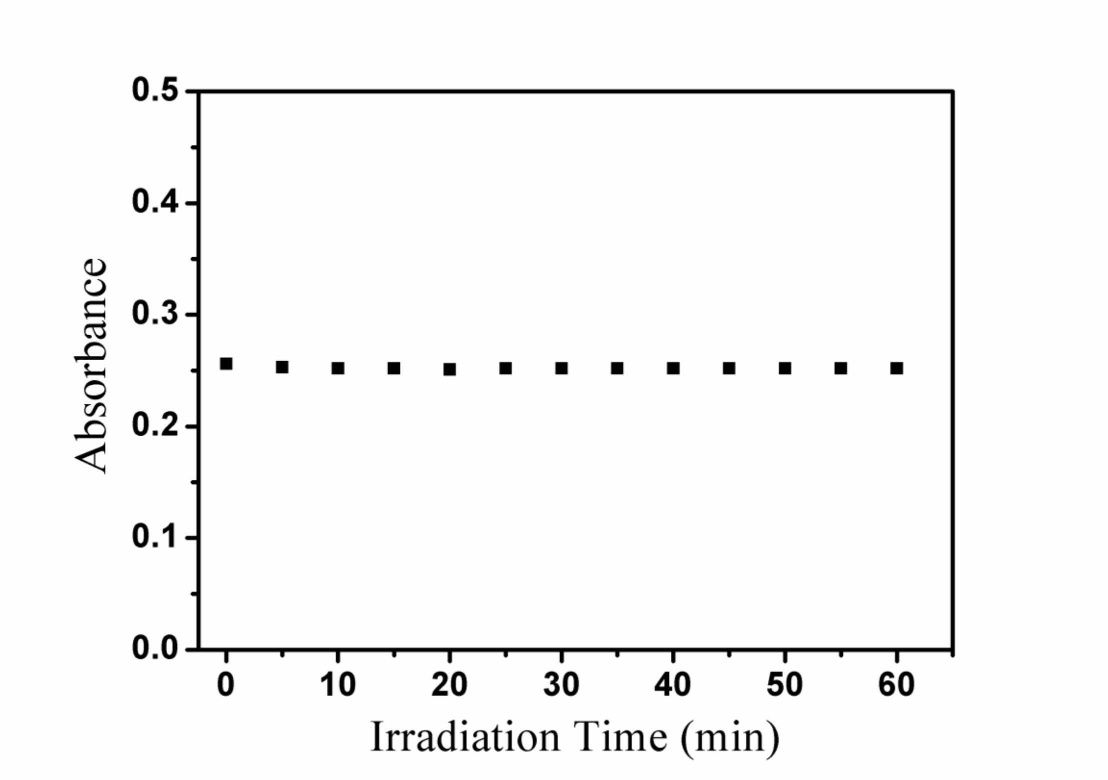

Figure S9. Optical absorbance at 808 nm versus the irradiation time.

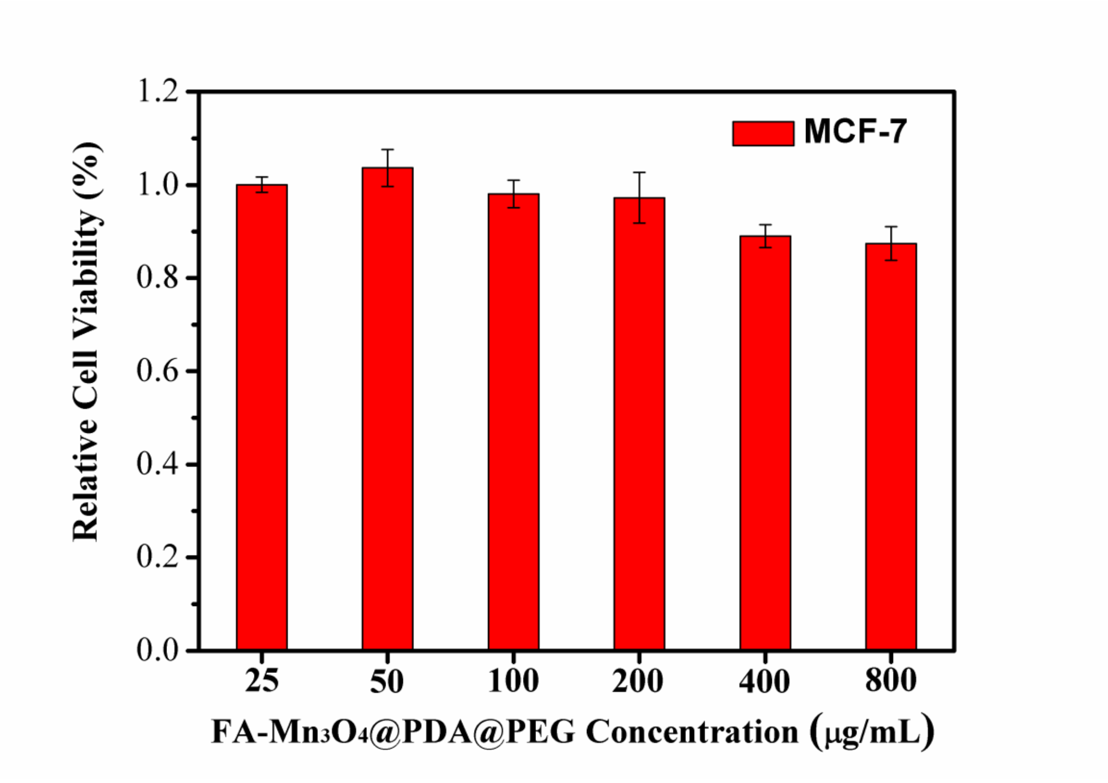

Figure S10. Cell viability of MCF-7 cells after incubation with increased concentrations of FA-Mn_3O_4@PDA@PEG.

References

[R1] M. H. Oh, T. Yu, S. H. Yu, B. Lim, K. T. Ko, M. G. Willinger, D. H. Seo, B. H. Kim, M. G. Cho, J. H. Park, K. Kang, Y. E. Sung, N. Pinna and T. Hyeon, *Science* **2013**, *340*, 964.

[R2] F. Liu, X. He, Z. Lei, L. Liu, J. Zhang, H. You, H. Zhang and Z. Wang, *Adv. Healthcare Mater*. **2015**, *4*, 559.

[R3] L. Zhang, L. Zeng, Y. Pan, S. Luo, W. Ren, A. Gong, X. Ma, H. Liang, G. Lu and A. Wu, *Biomaterials* **2015**, *44*, 82.

[R4] T. Kim, E. Momin, J. Choi, K. Yuan, H. Zaidi, J. Kim, M. Park, N. Lee, M. T. McMahon, A. Quinones-Hinojosa, J. W. Bulte, T. Hyeon and A. A. Gilad, *J. Am. Chem. Soc.* **2011**, *133*, 2955.

[R5] C. C. Huang, N. H. Khu and C. S. Yeh, *Biomaterials* **2010**, *31*, 4073.

[R6] M. Park, N. Lee, S. H. Choi, K. An, S. H. Yu, J. H. Kim, S. H. Kwon, D. Kim, H. Kim, S. Baek, T. Y. Ahn, O. K. Park, J. S. Son, Y. E. Sung, Y. W. Kim, Z. Wang, N. Pinna and T. Hyeon, *Chem. Mater.* **2011**, *23*, 3318.

[R7] H. Yang, Y. Zhuang, H. Hu, X. Du, C. Zhang, X. Shi, H. Wu and S. Yang, *Adv. Funct. Mater.* **2010**, *20*, 1733–1741.

[R8] J. Shin, R. M. Anisur, M. K. Ko, G. H. Im, J. H. Lee and I. S. Lee, *Angew. Chem. Int. Ed.* **2009**, *48*, 321.

[R9] K. M. L. Taylor, W. J. Rieter and W. Lin, *J. Am. Chem. Soc.* **2008**, *130*, 14358.

[R10] A. A. Gilad, P.Walczak, M. T. McMahon, H. B. Na, J. H. Lee, K. An, T. Hyeon, P. C.M. van Zijl and J. W. M. Bulte, *Magn. Reson. Med.* **2008**, *60*, 1.

[R11] Y. Chen, H. Chen, S. Zhang, F. Chen, S. Sun, Q. He, M. Ma, X. Wang, H. Wu, L. Zhang, L. Zhang and J. Shi, *Biomaterials* **2012**, *33*, 2388.

[R12] J. Xiao, X. M. Tian, C. Yang, P. Liu, N. Q. Luo, Y. Lian, H. B. Li, D. H. Chen,

C. X. Wang, L. Li and G. W. Yang, *Sci. Rep.* **2013**, *3*, 3424.

[R13] J. Huang, J. Xie, K. Chen, L. Bu, S. Lee, Z. Cheng, X. Li and X. Chen, *Chem. Commun.* **2010,** *46*, 6684.

[R14] N. Chen, C. Shao, Y. Qu, S. Li, W. Gu, T. Zheng, L. Ye and C. Yu, *ACS Appl. Mater. Interfaces* **2014**, *6*, 19850.

[R15] K. An, M. Park, J. H. Yu, H. B. Na, N. Lee, J. Park, S. H. Choi, I. C. Song, W. K. Moon and T. Hyeon, *Eur. J. Inorg. Chem.* **2012**, 2148.

[R16] H. Hu, A. Dai, J. Sun, X. Li, F. Gao, L. Wu, Y. Fang, H. Yang, L. An, H. Wu and S. Yang, *Nanoscale*, **2013**, *5*, 10447.

[R17] B. Y. W. Hsu, M. Wang, Y. Zhang, V. Vijayaragavan, S. Y. Wong, A. Y.C. Chang, K. K. Bhakoo, X. Li and J. Wang, *Nanoscale*, **2014**, *6*, 293.

[R18] S. Zhang, X. Qian, L. Zhang, W. Peng and Y. Chen, *Nanoscale*, **2015**, *7*, 7632.

[R19] A. Z. Abbasi, P. Prasad, P. Cai, C. He, W. D. Foltz, M. A. Amini, C. R. Gordijo, A. M. Rauth and X. Y. Wu, *J. Control Release* **2015**, *209*, 186.

[R20] B. Y. W. H, M. Ng, Y. Zhang, S. Y. Wong, K. Bhakoo, X. Li and J. Wang, *Adv. Funct. Mater.* **2015**, *25*, 5269.

[R21] M. Nafiujjaman, M. Nurunnabi, S. Kang, G. R. Reeck, H. A. Khand and Y. Lee, *J. Mater. Chem. B*, **2015**, *3*, 5815.
